# Predictors of radiographic erosion and joint space narrowing progression in patients with early rheumatoid arthritis: a cohort study

**DOI:** 10.1186/s13075-020-02413-7

**Published:** 2021-01-14

**Authors:** Emil Rydell, Kristina Forslind, Jan-Åke Nilsson, Magnus Karlsson, Kristina E. Åkesson, Lennart T. H. Jacobsson, Carl Turesson

**Affiliations:** 1 Rheumatology, Department of Clinical Sciences Malmö, Lund University, Jan Waldenströms gata 1B, SE-205 02 Malmö, Sweden; 2 Department of Rheumatology, Skåne University Hospital, Malmö, Sweden; 3 Rheumatology, Department of Clinical Sciences Lund, Lund University, Lund, Sweden; 4 Spenshult Research and Development Centre, Halmstad, Sweden; 5 Clinical and Molecular Osteoporosis Research Unit, Department of Clinical Sciences Malmö, Lund University, Malmö, Sweden; 6 Department of Orthopaedics, Skåne University Hospital, Malmö, Sweden; 7 Department of Rheumatology and Inflammation Research, Sahlgrenska Academy at Gothenburg University, Göteborg, Sweden

**Keywords:** Rheumatoid arthritis, Radiographic progression, Erosion, Joint space narrowing, Joint damage

## Abstract

**Background:**

Radiographic damage in rheumatoid arthritis (RA) includes erosions and joint space narrowing (JSN). Different mechanisms may underlie their development. The objective of this study was to evaluate predictors of these entities separately.

**Methods:**

Consecutive early RA patients (symptom duration ≤12 months) from a defined area (Malmö, Sweden) recruited during 1995–2005 were investigated. Radiographs of hands and feet were scored by a trained reader according to the modified Sharp-van der Heijde score. Fat mass and lean mass distribution were measured at baseline using dual energy x-ray absorptiometry. Potential predictors of erosion and JSN progression from inclusion to the 5-year follow-up were evaluated.

**Results:**

Two hundred and thirty-three patients were included. Radiographs at baseline and 5 years were available for 162 patients. The median (interquartile) progression of erosion and JSN scores were 4 (0–8) and 8 (1–16), respectively. Rheumatoid factor (RF) was a robust significant predictor of both erosion and JSN score progression. In adjusted analyses, anti-CCP antibodies predicted erosions while the erythrocyte sedimentation rate was predictive of both outcomes. Smoking and high baseline disease activity (DAS28 > 5.1) predicted progression of erosions. Baseline erosion score was associated with progression of both erosion and JSN progression, while baseline JSN score was predictive only of the progression of JSN. Overweight/obesity (BMI ≥ 25 kg/m^2^) was a significant negative predictor of JSN score progression (*β* = − 0.14, *p* = 0.018, adjusted for RF, age, baseline JSN score) also when additionally adjusting for ever smoking (*p* = 0.041). Among female patients, this effect was observed in those of estimated post-menopausal age (> 51 years), but not in younger women. The truncal to peripheral fat ratio was associated with less JSN score progression in women, but not in men.

**Conclusions:**

Overweight RA patients had less JSN progression, independent of smoking status. This effect was seen in particular among older women (mainly post-menopausal), but not younger. Truncal fat was associated with less JSN progression in female patients. Smoking predicted erosion progression, and erosions may precede JSN. BMI and fat distribution may influence cartilage damage in early RA and might be related to hormonal factors.

## Introduction

In patients with rheumatoid arthritis (RA), detection of early joint damage by radiography is prognostic and has been previously shown to identify patients more prone to further damage progression [1–5]. The presence of autoantibodies such as rheumatoid factor (RF) and anti-citrullinated protein antibodies (ACPAs), higher disease activity measures, and levels of systemic inflammation are considered other established risk factors for worse radiological outcomes [6]. Increased cartilage turnover, measured as serum levels of the cartilage oligomeric matrix protein (COMP), may also predict progression of joint damage [7–10]. There are conflicting data on the impact of patient age and sex [6], while for cigarette smoking and body constitution there is mounting evidence suggesting important associations with radiographic progression. In previously published data, we have shown that smoking is associated with more radiographic progression and high body mass index (BMI) is associated with less radiographic progression in early RA [11]. Similar results have been presented by others [12–16]. Possible mechanisms for the effects of BMI on radiographic progression have been suggested to be hormonally related, where adipokines have gained particular interest [17, 18]. Whether the relationship between BMI and joint damage is age dependent has not been investigated. RA-related joint damage includes erosions and cartilage destruction, with the latter causing joint space narrowing (JSN). Despite that separate scores of these are included in the commonly used radiographic scoring methods [19], most previous studies have evaluated only combined total scores of the radiographic damage. Whether the development and progression of erosions and JSN represent separate underlying mechanisms in the process of destructive arthritis in RA is unclear [20]. The purpose of this study was to investigate how patient characteristics, smoking status, disease activity measures, anthropometrics, and body composition measures relate to subsequent progression of erosions and JSN separately, in patients with early RA. We also wanted to study if the significance of anthropometrics and body composition on radiographic progression differed among older compared to younger women.

## Methods

### Patients

An inception cohort of 233 consecutive patients with early RA was investigated. The catchment area was the city of Malmö, Sweden (population 259,579 in 2000). Patients were recruited from the rheumatology outpatient clinic of Skåne University Hospital Malmö, the only hospital serving the city, or from the four rheumatologists in private practice in the area, between 1995 and 2005.

The patients were diagnosed with RA by a specialist in rheumatology, fulfilled the 1987 American College of Rheumatology (ACR) classification criteria for RA [21] and had duration of symptoms ≤ 12 months at the time of inclusion (baseline). There were no additional exclusion criteria.

Results on predictors for radiographic progression using Sharp-van der Heijde (SHS) total score in this present cohort have been reported previously [11].

### Clinical assessment

Patients were followed according to a structured program with evaluations at baseline, 1 year, and 5 years. The same rheumatologist performed all the clinical examinations. Patient characteristics and disease activity parameters were recorded, and radiographs of hands and feet were obtained. The presence of erosions (present vs. absent) was determined by a radiologist as part of standard clinical practice. Disability was assessed using the Swedish version of the health assessment questionnaire (HAQ) [22]. Visual analogue scales (VAS) were used to evaluate the patient’s global assessment of disease activity and patient’s assessment of pain. All patients were managed according to usual care with no pre-specified protocol for anti-rheumatic treatment. The patients were included before the current practice of treat to target [23] was implemented, and before early treatment with biologic disease-modifying anti-rheumatic drugs (DMARDs) came into widespread use. Information on height, weight, and smoking history (current/previous/never) was collected at inclusion through a self-administered questionnaire. The time from symptom onset to first start of DMARD treatment was assessed based on a review of medical records.

Data on treatment with biologic DMARDs at any time during the study period was obtained through linkage to a regional biologics register [24].

### Total body scan

Total and regional fat mass, lean mass, and bone mineral content were measured at baseline using dual energy x-ray absorptiometry (DXA) (Lunar DPX-L equipment, 1.3z Lunar®, Madison, WI, USA). Fat mass was reported for total body, trunk, and arms and legs, whereas lean mass was reported for total body and arms and legs, and bone mineral content only for total body. Truncal to peripheral fat ratio was calculated by dividing the truncal fat mass with the fat mass of arms and legs. Fat mass index was calculated as total body fat mass (BFM) divided by height-squared. Fat free mass index was calculated as the sum of total body lean mass and bone mineral content, divided by height-squared. Fat mass lean mass index was calculated as BFM divided by total body lean mass. The precision of our DXA apparatus in vivo was evaluated by double measurements after repositioning in 14 healthy adults, as previously reported [25], with coefficients of variation for total body bone mineral density (BMD) 0.4%, lumbar spine BMD 0.5%, femoral neck BMD 1.6%, BFM 4.1%, and total lean mass 0.6%.

### Laboratory investigations

IgM RF was analyzed using ELISA, which was calibrated against the World Health Organization (WHO) RF reference preparation. Anti-CCP antibodies were analyzed using the Quanta Lite CCP IgG ELISA (INOVA Diagnostics, US). Erythrocyte sedimentation rate (ESR) and C-reactive protein (CRP) were assessed according to standard methods at the Department of Clinical Chemistry, Malmö University Hospital. Serum COMP concentrations were determined using a sandwich ELISA (AnaMar, Lund, Sweden).

### Radiographic assessment

Radiographs of hands and feet were scored in chronological order according to the modified Sharp-van der Heijde score (SHS) [26]. One trained reader (KF, co-author), blinded to the clinical data, scored the radiographs for SHS including subscores of erosion and JSN score. For total SHS the maximum score for hands and feet are 448, for erosion score 280 and for JSN score 168. The intra-class correlation coefficient (ICC) from two readings with 2-week intervals of a subset of the cohort (*n* = 30) was 0.97. Based on the excellent ICC, a single reading was performed. The primary outcome was progression of erosions score and JSN score up to the 5-years follow-up.

### Statistical analysis

Potential associations between each individual baseline variable with the amount of progression of erosion and JSN scores from inclusion to the 5 year follow-up were assessed using linear regression analyses. Six patients had discreet negative progression of erosions indicated by a 1–2-unit decrease in erosions score over 5 years. One patient had negative progression of JSN score indicated by a 2-unit decrease. This might represent healing or small reading errors [27]. The outcome variables were logarithmically transformed because of non-normal distribution of residuals. To allow for logarithmic computation without censoring individuals with progression of negative or zero-values, the smallest possible constant was added.

Co-variates for the multivariate models were chosen based on the literature and the unadjusted analyses. Due to co-linearity, we did not include both RF and anti-CCP. RF was chosen over anti-CCP due to the smaller number of patients with missing data for RF. The final multivariate analyses for erosion progression were adjusted for RF and baseline erosion score, and analyses for JSN progression were adjusted for RF, age and baseline JSN score. Due to the known association between smoking and BMI, models including BMI were additionally adjusted for smoking and vice versa.

During part of the study period, high sensitivity CRP analysis was not available and CRP values between 0 and 9 mg/l was reported by the laboratory as < 9 mg/l. In the regression models, CRP was therefore included as a dichotomized variable, i.e., above versus below median (9 mg/l) at inclusion. Current smoking, previous smoking, and ever smoking (current and previous) were each compared to the reference category, never smoking.

BMI was included as a continuous variable. Furthermore, individuals fulfilling the WHO criteria for overweight or obesity (≥ 25 kg/m^2^), overweight (25–29.99 kg/m^2^), or obesity (≥ 30 kg/m^2^), were compared to those with normal BMI (18.5–24.99 kg/m^2^). Three patients with BMI < 18.5 kg/m^2^ were excluded from analyses of BMI categories.

Possible interaction effects for anthropometrics and body composition measures with age category (> 51 years vs ≤ 51 years of age) were evaluated in women. This cut-off was chosen to represent an estimated menopausal age, as reported in Swedish women [28]. For co-variates with significant interaction effects, results from analyses stratified by age category are presented (Table 7). Statistical analysis was performed using IBM SPSS Statistics version 25.0, Armonk, NY, IBM Corp.

## Results

### Patient characteristics

In this study, 233 consecutive early RA patients [median symptom duration 7 months; interquartile range (IQR) 5–10] were included. Eighty-six percent of patients with early RA had detectable radiographic progression of joint damage over the first 5 years of follow-up, with numerically more progression of JSN than erosion scores. Characteristics at baseline and at the 5-year follow-up in patients with available radiographic data, as well as those with additional body composition data, are shown in Table 1. Disease parameters and treatment at baseline and at 5 years in these groups are shown in Table 2. Seventeen percent (*n* = 40) of all patients in the full cohort were treated with a biologic DMARD at some time during the first 5 years.

**Table 1:** Baseline patient characteristics

	With radiographic data available from baseline and 5 years (***n*** = 162)	With radiographic data available from baseline and 5 years and DXA at baseline (***n*** = 97)
**Demographics and history**		
Female sex, *n* (%)	114 (70)	72 (74)
Age (years)	62 (52–70)	63 (52–71)
Symptom duration (months)	7 (5–10)	8 (6–10)
Time to first DMARD (months)^a^	5 (3–7)	5 (4–7)
RF positive, *n* (%)	105 (65)	56 (58)
Anti-CCP positive, *n* (%)	83 (59)	55 (59)
**Cigarette smoking status**		
Current smokers, *n* (%)	49 (32)	32 (33)
Previous smokers, *n* (%)	51 (33)	33 (34)
Never smokers, *n* (%)	55 (36)	32 (33)
**Anthropometrics**		
BMI (kg/m^2^)	25 (23–28)	25 (23–27)
Obese, *n* (%)^b^	19 (12)	6 (6)
Overweight, *n* (%)^b^	69 (45)	46 (48)
Normal BMI, *n* (%)^b^	66 (43)	43 (45)
**Body composition**		
Truncal to peripheral fat ratio	1.08 (0.89–1.27)	1.08 (0.89–1.27)
Total body fat percentage	35 (27–40)	35 (27–40)
Fat mass index	8.64 (6.07–10.52)	8.64 (6.07–10.52)
Fat free mass index	15.60 (14.90–16.97)	15.60 (14.90–16.97)
Fat mass lean mass index	0.56 (0.39–0.72)	0.56 (0.39–0.72)

Median (IQR) given unless otherwise stated

Missing numbers (radiology only group/radiology + DXA group): Symptom duration = 1/1, time to DMARD = 15/9, cigarette smoking status = 7/-, BMI = 5/1

*DMARD* disease-modifying anti-rheumatic drug, *RF* rheumatoid factor, *Anti-CCP* anti-citrullinated peptide antibodies, *BMI* body mass index, *IQR* interquartile range

^a^Duration from RA symptom onset to start of first DMARD

^b^Definitions based on BMI: obese ≥ 30 kg/m^2^, overweight 25–29.99 kg/m^2^, normal 18.5–24.99 kg/m^2^. Three patients with BMI < 18.5 kg/m^2^ were excluded from analyses of BMI categories

**Table 2:** Treatment and disease parameters at baseline and 5 years

	With radiographic data available from baseline and 5 years (***n*** = 162)		With radiographic data available from baseline and 5 years and DXA at baseline (***n*** = 97)	
	Baseline	5 years	Baseline	5 years
**Treatment**				
DMARD (any), *n* (%)	138 (85)	122 (75)	77 (79)	71 (73)
MTX, *n* (%)	85 (52)	97 (60)	33 (34)	52 (53)
MTX dose (mg/week)	10.0 (7.5–10.0)	10.0 (7.5–15.0)	7.5 (5.0–10.0)	7.5 (5.0–15.0)
Antimalarials, *n* (%)	47 (29)	17 (10)	39 (40)	15 (15)
Combination (≥ 2 cDMARDs)	3 (2)	14 (9)	4 (4)	10 (10)
Biologic, *n* (%)	0 (0)	26 (16)	0 (0)	9 (9)
Prednisolone, *n* (%)	60 (37)	47 (29)	29 (30)	32 (33)
Prednisolone dose (mg/day)	7.5 (5.0–15.0)	5.0 (2.5–5.0)	5.0 (5.0–10.0)	5.0 (2.5–7.5)
**Disease parameters**				
COMP, units/l	11 (9–14)	NR	11 (9–14)	NR
Modified Sharp-van der Heijde score (SHS)	2 (0–8)	17 (5–31)	1 (0–6)	16 (5–32)
Erosion score	0 (0–2)	5 (1–10)	0 (0–1)	5 (1–10)
Joint space narrowing (JSN) score	0 (0–6)	11 (3–22)	0 (0–5)	10 (3–21)
Progression of SHS from baseline (≥1 unit), n (%)	N/A	140 (86)	N/A	83 (86)
Progression of erosion score from baseline (≥1 unit), n (%)	N/A	122 (75)	N/A	71 (73)
Progression of JSN score from baseline (≥1 unit), n (%)	N/A	122 (75)	N/A	69 (71)
Erosions present, *n* (%)^a^	28 (17)	63 (39)	19 (20)	39 (40)
DAS28	4.7 (3.6–4.7)	3.5 (2.6–4.5)	4.6 (3.5–5.5)	3.5 (2.6–4.6)
Remission, *n* (%)^b^	12 (7.5)	39 (25)	8 (8)	24 (26)
Low disease activity, *n* (%)^b^	27 (16.8)	70 (45)	17 (18)	39 (42)
Moderate disease activity, *n* (%)^b^	75 (46.6)	63 (40)	45 (47)	39 (42)
High disease activity, *n* (%)^b^	59 (36.6)	23 (15)	34 (35)	15 (16)
HAQ	0.75 (0.38–1.25)	0.75 (0.13–1.12)	0.75 (0.25–1.13)	0.75 (0.13–1.25)
Swollen joint count (out of 28)	7 (5–11)	4 (2–7)	7 (4–11)	4 (2–8)
Tender joint count (out of 28)	4 (2–9)	1 (0–4)	4 (1–8)	1 (0–5)
ESR (mm/h)	22 (11–43)	15 (9–24)	22 (10–38)	15 (9–24)
CRP (mg/l)	9 (< 9–28)	3 (< 9–10)	< 9 (< 9–21)	< 9 (< 9–12)
Patient’s global assessment (VAS 0–100 mm)	46 (21–65)	37 (12–52)	46 (20–65)	40 (10–58)
Pain (VAS 0–100 mm)	40 (19–61)	29 (9–48)	40 (18–62)	31 (8–51)

Median (IQR) given unless otherwise stated

Missing numbers in patients with radiographic data, baseline/5 years: COMP = 20/-, Erosion present = 1/0, DAS28 = 1/6, HAQ = 1/2, 28-swollen joint count = 1/2, 28-tender joint count = 1/2, ESR = 1/5, CRP = 1/3, VAS-patient global health = 1/3, VAS-pain = 1/2

Missing numbers in patients with radiographic and DXA data, baseline/5 years: COMP = 1/-, Erosion present = 1/0, DAS28 = 1/4, HAQ = 1/2, 28-swollen joint count = 1/2, 28-tender joint count = 1/2, ESR = 1/4, CRP = 1/2, VAS-patient global health = 1/2, VAS-pain = 1/2

*DMARD* disease-modifying anti-rheumatic drug, *MTX* methotrexate, *COMP* cartilage oligomeric matrix protein, *NR* not reported, *SHS* modified Sharp-van der Heijde score, *JSN* joint space narrowing, *N/A* not applicable, *DAS28* disease activity score of 28 joints, *HAQ* health assessment questionnaire, *ESR* erythrocyte sedimentation rate, *CRP* C-reactive protein, *VAS* visual analogue scale, *IQR* interquartile range

^a^Determined by a radiologist as part of standard clinical practice

^b^DAS28-classifications: remission ≤ 2.6, low > 2.6 to ≤ 3.2, moderate > 3.2 to ≤ 5.1, high > 5.1

### Radiographic progression

Radiographic data was available for 217 patients at baseline and 171 at 5 years. One hundred and sixty-two patients had data from both time points. The mean progression of erosion and JSN scores from baseline to 5 years were 6.1 (*n* = 163, standard deviation (SD) = 8.8) and 11.2 (*n* = 162, SD = 12.8), respectively.

### Baseline predictors of erosion and JSN score progression

The results of analyses of associations between baseline variables and erosion and JSN score progression up to 5 years are shown in Tables 3 and 4, respectively. Sex was not a significant predictor of erosion or JSN score progression at 5 years in crude or adjusted analyses (Tables 3 and 4), while there was a trend for age as a predictor of JSN score progression (*p* = 0.051) (Table 4).

**Table 3:** Baseline predictors of erosion score progression up to 5 years

	Crude		Adjusted^**a**^	
	***β***	95% CI	***β***	95% CI
**Demographics**				
Male sex	0.08	− 0.03, 0.20	0.06	− 0.04, 0.17
Age (per SD)^b^	0.02	− 0.03, 0.08	0.02	− 0.03, 0.07
Time to first DMARD (per SD)^b^	0.01	− 0.04, 0.06	0.01	− 0.04, 0.05
**Smoking habits**				
Never smoker (reference)	0.00		0.00	
Current smoker	**0.17**	**0.04, 0.30**	0.12	− 0.00, 0.24
Previous smoker	**0.15**	**0.03, 0.27**	**0.12**	**0.01, 0.23**
Ever smoker	**0.16**	**0.05, 0.27**	**0.12**	**0.02, 0.22**
**Baseline disease parameters**				
RF positivity	**0.23**	**0.13, 0.34**	**0.24**^**c**^	**0.14, 0.34**^**c**^
Anti-CCP positivity	**0.23**	**0.12, 0.33**	**0.13**	**0.02, 0.24**
COMP titer (per SD)^b^	0.04	− 0.01, 0.10	0.04	− 0.01, 0.09
COMP > 12 units/l	0.10	− 0.01, 0.21	**0.12**	**0.02, 0.22**
Erosions present^d^	**0.21**	**0.08, 0.34**	**0.16**^**e**^	**0.03, 0.29**
Erosion score (per SD)^b^	**0.07**	**0.02, 0.12**	**0.08**^**e**^	**0.03, 0.12**
Joint space narrowing score (per SD)^b^	0.04	− 0.01, 0.09	0.04^**e**^	− 0.01, 0.08
DAS28 (per SD)^b^	**0.05**	**0.001, 0.11**	0.04	− 0.01, 0.08
Disease activity^d^				
Low/moderate (reference)	0.00		0.00	
High	**0.15**	**0.05, 0.25**	**0.11**	**0.01, 0.21**
HAQ (per SD)^b^	0.03	− 0.02, 0.08	0.03	− 0.02, 0.07
ESR (per SD)^b^	**0.11**	**0.06, 0.16**	**0.08**	**0.03, 0.12**
CRP below median (reference)	0.00		0.00	
CRP above median (> 9 mg/l)	**0.15**	**0.06, 0.25**	0.09	− 0.01, 0.18
Swollen joint count (per SD)^b^	0.03	− 0.02, 0.09	0.03	− 0.02, 0.08
Tender joint count (per SD)^b^	− 0.03	− 0.09, 0.03	− 0.02	− 0.07, 0.04
Patient’s global assessment (VAS; per SD)^b^	0.05	− 0.01, 0.10	0.03	− 0.02, 0.08
Pain (VAS; per SD)^b^	0.02	− 0.03, 0.08	0.02	− 0.02, 0.07

Numbers in bold indicate significance of *p* ≤ 0.05

*CI* confidence interval, *SD* standard deviation, *DMARD* disease-modifying anti-rheumatic drug, *RF* rheumatoid factor, *Anti-CCP* anti-citrullinated peptide antibodies, *COMP* cartilage oligomeric matrix protein, *DAS28* disease activity score of 28 Joints, *HAQ* health assessment questionnaire, *ESR* erythrocyte sedimentation rate, *CRP* C-reactive protein, *VAS* visual analogue scale

^a^Adjusted for RF and baseline erosion score

^b^SD: age 14 years; time to first DMARD 5.8 months; COMP 3.6 units/l; erosion score 3; JSN score 8; DAS28 1.4; HAQ 0.64; ESR 26 mm/h; swollen joint count 4.8; tender joint count 5.8; patient’s global assessment 26; pain 26

^c^Adjusted for baseline erosion score

^d^For definitions see Table 1

^e^Adjusted only for RF

**Table 4:** Baseline predictors of joint space narrowing score progression up to 5 years

	Crude		Adjusted^**a**^	
	***β***	95% CI	***β***	95% CI
**Demographics**				
Male sex	0.00	− 0.13, 0.13	− 0.02	− 0.14, 0.11
Age (per SD)^b^	0.06	− 0.00, 0.12	0.03^c^	− 0.03, 0.10^c^
Time to first DMARD (per SD)^b^	− 0.01	− 0.07, 0.04	0.00	− 0.06, 0.05
**Smoking habits**				
Never smoker (reference)	0.00		0.00	
Current smoker	0.06	− 0.08, 0.21	0.10	− 0.05, 0.25
Previous smoker	0.03	− 0.12, 0.17	0.03	− 0.10, 0.17
Ever smoker	0.05	− 0.08, 0.17	0.06	− 0.06, 0.19
**Baseline disease parameters**				
RF positivity	**0.18**	**0.06, 0.31**	**0.18**^**d**^	**0.06, 0.30**^**d**^
Anti-CCP positivity	**0.17**	**0.05, 0.30**	0.09	− 0.04, 0.23
COMP titer (per SD)^b^	0.04	− 0.02, 0.10	0.04	− 0.03, 0.10
COMP > 12 units/l	0.08	− 0.05, 0.20	0.08	− 0.05, 0.20
Erosions present^e^	**0.19**	**0.03, 0.35**	0.13^f^	− 0.03, 0.29^f^
Erosion score (per SD)^b^	**0.12**	**0.06, 0.17**	**0.11**^**f**^	**0.05, 0.16**^**f**^
Joint space narrowing score (per SD)^b^	**0.11**	**0.06, 0.17**	**0.10**^**f**^	**0.04, 0.16**^**f**^
DAS28 (per SD)^b^	0.02	− 0.05, 0.08	0.01	− 0.05, 0.06
Disease activity^e^				
Low/moderate (reference)	0.00		0.00	
High	0.10	− 0.02, 0.23	0.08	− 0.04, 0.20
HAQ (per SD)^b^	0.02	− 0.04, 0.08	0.03	− 0.02, 0.09
ESR (per SD)^b^	**0.09**	**0.03, 0.15**	**0.06**	**0.003, 0.12**
CRP below median (reference)	0.00		0.00	
CRP above median (> 9 mg/l)	**0.13**	**0.01, 0.25**	0.07	− 0.05, 0.19
Swollen joint count (per SD)^b^	0.00	− 0.06, 0.06	0.00	− 0.06, 0.06
Tender joint count (per SD)^b^	**− 0.07**	**− 0.14, − 0.003**	− 0.05	− 0.13, 0.02
Patient’s global assessment (VAS; per SD)^b^	0.03	− 0.03, 0.09	0.02	− 0.04, 0.08
Pain (VAS; per SD)^b^	0.00	− 0.06, 0.06	0.02	− 0.04, 0.08

Numbers in bold indicate significance of *p* ≤ 0.05

*CI* confidence interval, *SD* standard deviation, *DMARD* disease-modifying anti-rheumatic drug, *RF* rheumatoid factor, *Anti-CCP* anti-citrullinated peptide antibodies, *COMP* cartilage oligomeric matrix protein, *DAS28* disease activity score of 28 joints, *HAQ* health assessment questionnaire, *ESR* erythrocyte sedimentation rate, *CRP* C-reactive protein, *VAS* visual analogue scale, *JSN* joint space narrowing

^a^Adjusted for RF, age and baseline JSN score

^b^SD: age 15 years; time to first DMARD 5.8 months; COMP 3.6 units/l; erosion score 3; JSN score 8; DAS28 1.4; HAQ 0.64; ESR 26 mm/h; swollen joint count 4.9; tender joint count 5.8; patient’s global assessment 26; pain 26

^c^Adjusted for RF and baseline JSN score

^d^Adjusted for age and baseline JSN score

^e^For definitions see Table 1

^f^Adjusted for RF and age

### Smoking habits

Significant associations with erosion score progression from baseline up to 5 years were observed for ever smoking and previous smoking in crude and adjusted models (Table 3), with a trend for current smoking [adjusted *β* = 0.12; p = 0.051]. In analyses additionally adjusted for BMI, current smoking [*β* = 0.13 (95% CI 0.01, 0.26)] and ever smoking [*β* = 0.12 (95% CI 0.02, 0.22)] were both predictive of erosion score progression over 5 years, with a trend for previous smoking [*β* = 0.11; *p* = 0.055]. Smoking status was not predictive of JSN score progression (Table 4).

### Anthropometrics and body composition

Overweight or obese patients had a reduced risk of JSN score progression up to 5 years compared to those with normal BMI (Table 5). The presence of overweight/obesity (BMI ≥25 kg/m^2^) was associated with a significantly reduced risk of JSN score progression up to 5 years in analyses adjusted for RF, age, and JSN score at baseline (Table 5), and also when additionally adjusting for ESR [*β* = − 0.13 (95% CI − 0.25, − 0.02)], or ever smoking [*β* = − 0.13 (95% CI − 0.25, − 0.01)]. In adjusted analyses stratified by sex, the negative association between overweight/obesity reached statistical significance in women [*β* = − 0.15 (95% CI − 0.30, − 0.01)], with a similar estimate in men [*β* = − 0.13 (95% CI − 0.36, 0.09)]. The effect of overweight/obesity on JSN score progression up to 5 years was mainly observed in RF positive [*β* = − 0.22 (95% CI − 0.38, − 0.07)] and not RF negative [*β* = − 0.01 (95% CI − 0.21, 0.19)] patients, and similarly stratified for anti-CCP status [positive: *β* = − 0.19 (95% CI − 0.37, − 0.02), negative: *β* = − 0.06 (95% CI − 0.26, 0.15)].

**Table 5:** Baseline anthropometrics and body composition as predictors of joint space narrowing progression over 5 years

	All				Women				Men			
	Crude		Adjusted^**a**^		Crude		Adjusted^**a**^		Crude		Adjusted^**a**^	
	***β***	95% CI	***β***	95% CI	***β***	95% CI	***β***	95% CI	***β***	95% CI	***β***	95% CI
**Anthropometrics**												
BMI (per SD)^b^	− 0.03	− 0.09, 0.03	− 0.04	− 0.10, 0.02	− 0.03	− 0.11, 0.04	− 0.04	− 0.11, 0.03	− 0.02	− 0.14, 0.10	− 0.05	− 0.16, 0.07
Normal BMI^c^ (reference)	0.00		0.00		0.00		0.00		0.00		0.00	
Obese^c^	− 0.14	− 0.34, 0.06	− 0.14	− 0.31, 0.04	− 0.18	− 0.41, 0.06	− 0.18	− 0.37, 0.02	− 0.07	− 0.46, 0.32	− 0.07	− 0.48, 0.34
Obese or overweight^c^	**− 0.14**	**− 0.26, − 0.01**	**− 0.14**	**− 0.26, − 0.03**	− 0.15	− 0.30, 0.01	**− 0.15**	**− 0.30, − 0.01**	− 0.11	− 0.34, 0.12	− 0.13	− 0.36, 0.09
Overweight^c^	− 0.13	− 0.27, 0.00	**− 0.15**	**− 0.28, − 0.02**	− 0.14	− 0.31, 0.03	− 0.15	− 0.31, 0.01	− 0.12	− 0.38, 0.14	− 0.16	− 0.41, 0.09
**Body composition**												
Truncal to peripheral fat ratio (per SD)^b^	− 0.02	− 0.10, 0.06	− 0.07	− 0.15, 0.01	− 0.09	− 0.21, 0.03	**− 0.14**	**− 0.25, − 0.02**	0.05	− 0.12, 0.21	0.03	− 0.15, 0.22
Total body fat percentage (per SD)^b^	− 0.04	− 0.12, 0.05	− 0.04	− 0.12, 0.04	− 0.03	− 0.15, 0.09	− 0.06	− 0.17, 0.06	− 0.04	− 0.28, 0.21	− 0.16	− 0.40, 0.08
Fat mass index (per SD)^b^	− 0.05	− 0.13, 0.04	− 0.06	− 0.14, 0.02	− 0.05	− 0.15, 0.06	− 0.06	− 0.16, 0.04	− 0.04	− 0.28, 0.20	− 0.17	− 0.40, 0.06
Fat free mass index (per SD)^b^	− 0.02	− 0.10, 0.07	− 0.03	− 0.12, 0.05	− 0.15	− 0.33, 0.04	− 0.12	− 0.30, 0.06	− 0.02	− 0.20, 0.16	− 0.06	− 0.23, 0.11
Fat mass lean mass index (per SD)^b^	− 0.04	− 0.12, 0.05	− 0.04	− 0.12, 0.04	− 0.03	− 0.14, 0.08	− 0.05	− 0.16, 0.06	− 0.07	− 0.39, 0.26	− 0.21	− 0.52, 0.10

Numbers in bold indicate significance of p ≤ 0.05

*CI* confidence interval, *BMI* body mass index, *SD* standard deviation, *RF* rheumatoid factor, *JSN* joint space narrowing

^a^Adjusted for RF, age, and baseline JSN score

^b^SD: BMI 4.0 kg/m^2^; truncal to peripheral fat ratio 0.27; total body fat percentage 8.6; fat mass index 3.0; fat free mass index 1.9; fat mass lean mass index 0.2

^c^For definitions see Table 1

There were no major differences in baseline CRP, ESR, DAS28, or smoking status across categories of BMI (data not shown). No significant associations were observed for BMI categories and erosion score progression (Table 6).

**Table 6:** Baseline anthropometrics and body composition as predictors of erosion progression over 5 years

	All				Women				Men			
	Crude		Adjusted^**a**^		Crude		Adjusted^**a**^		Crude		Adjusted^**a**^	
	***β***	95% CI	***β***	95% CI	***β***	95% CI	***β***	95% CI	***β***	95% CI	***β***	95% CI
**Anthropometrics**												
BMI (per SD)^b^	0.01	− 0.05, 0.06	0.01	− 0.04, 0.06	0.02	− 0.05, 0.08	0.02	− 0.04, 0.09	− 0.02	− 0.13, 0.08	− 0.04	− 0.14, 0.06
Normal BMI (reference)^c^	0.00		0.00		0.00		0.00		0.00		0.00	
Obese^c^	− 0.03	− 0.21, 0.15	− 0.04	− 0.21, 0.12	− 0.06	− 0.28, 0.17	− 0.05	− 0.26, 0.15	− 0.01	− 0.33, 0.32	− 0.03	− 0.35, 0.28
Obese or overweight^c^	− 0.05	− 0.16, 0.06	− 0.03	− 0.13, 0.08	− 0.04	− 0.17, 0.09	0.01	− 0.11, 0.13	− 0.09	− 0.29, 0.11	− 0.10	− 0.29, 0.09
Overweight^c^	− 0.06	− 0.18, 0.06	− 0.03	− 0.14, 0.08	− 0.04	− 0.18, 0.11	0.02	− 0.12, 0.15	− 0.12	− 0.33, 0.10	− 0.13	− 0.33, 0.08
**Body composition**												
Truncal to peripheral fat ratio (per SD)^b^	0.03	− 0.04, 0.10	0.01	− 0.06, 0.08	− 0.01	− 0.12, 0.09	− 0.03	− 0.13, 0.07	− 0.03	− 0.17, 0.10	− 0.03	− 0.14, 0.08
Total body fat percentage (per SD)^b^	− 0.02	− 0.09, 0.05	− 0.02	− 0.09, 0.04	0.05	− 0.05, 0.15	0.03	− 0.07, 0.13	− 0.01	− 0.21, 0.19	− 0.05	− 0.22, 0.12
Fat mass index (per SD)^b^	− 0.01	− 0.08, 0.07	− 0.02	− 0.09, 0.05	0.03	− 0.06, 0.12	0.01	− 0.07, 0.10	0.03	− 0.17, 0.22	− 0.02	− 0.19, 0.15
Fat free mass index (per SD)^b^	0.07	− 0.01, 0.14	0.05	− 0.02, 0.12	− 0.05	− 0.21, 0.11	− 0.04	− 0.19, 0.11	0.09	− 0.05, 0.23	0.08	− 0.04, 0.19
Fat mass lean mass index (per SD)^b^	− 0.02	− 0.09, 0.06	− 0.02	− 0.09, 0.05	0.05	− 0.05, 0.14	0.03	− 0.07, 0.12	− 0.03	− 0.30, 0.24	− 0.08	− 0.30, 0.15

Numbers in bold indicate significance of *p* ≤ 0.05

*CI* confidence interval, *BMI* body mass index, *SD* standard deviation, *RF* rheumatoid factor

^a^Adjusted for RF and baseline erosion score

^b^SD: BMI 4.0 kg/m^2^; truncal to peripheral fat ratio 0.27; total body fat percentage 8.6; fat mass index 3.0; fat free mass index 1.8; fat mass lean mass index 0.2

^c^For definitions see Table 1

Body composition measures and its relation to JSN and erosion score progression over 5 years are shown in Tables 5 and 6. For truncal to peripheral fat ratio, a significant negative association with JSN score progression was observed in the adjusted analysis in women [*β* = − 0.14 (95% CI − 0.25, − 0.02)], but not in men [*β* = 0.03 (95% CI − 0.15, 0.22)]. No associations were observed for total body fat percentage, fat mass index, fat free mass index, or fat mass lean mass index with JSN score progression (Table 5) and neither body composition variables with erosion score progression (Table 6).

To test if age had an influence on the effects seen for overweight/obesity and truncal to peripheral fat ratio with JSN score progression, female patients were categorized as > 51 years and ≤ 51 years of age. In the analyses of predictors of progression of JSN score, there were significant interactions between age category and overweight/obesity (*p* = 0.02), obesity (*p* = 0.03), and overweight (*p* = 0.047), in women. In analyses stratified for > 51 years of age, the presence of overweight/obesity had significant negative associations with JSN progression over 5 years in the older women, but not in younger (Table 7).

**Table 7:** Baseline anthropometrics as predictors of JSN progression over 5 years in women, stratified by age

	> 51 years				≤ 51 years			
	Crude		Adjusted^**a**^		Crude		Adjusted^**a**^	
	***β***	95% CI	***β***	95% CI	***β***	95% CI	***β***	95% CI
BMI (per SD)^b^	− 0.07	− 0.15, 0.01	− 0.06	− 0.14, 0.02	0.06	− 0.05, 0.16	− 0.04	− 0.16, 0.08
Normal BMI (reference)^c^								
Obese^c^	**− 0.34**	**− 0.62, − 0.06**	− 0.24	− 0.50, 0.02	0.14	− 0.24, 0.51	− 0.22	− 0.66, 0.23
Obese or overweight^c^	**− 0.27**	**− 0.44, − 0.09**	**− 0.21**	**− 0.38, − 0.04**	0.13	− 0.16, 0.43	− 0.04	− 0.36, 0.27
Overweight^c^	**− 0.26**	**− 0.44, − 0.07**	**− 0.20**	**− 0.38, − 0.03**	0.13	− 0.24, 0.50	0.01	− 0.36, 0.38

Numbers in bold indicate significance of *p* ≤ 0.05

*CI* confidence interval, *BMI* body mass index, *SD* standard deviation, *RF* rheumatoid factor, *JSN* joint space narrowing

^a^Adjusted for RF, age and baseline JSN score

^b^SD: BMI (> 51 years) 3.7 kg/m^2^; BMI (≤ 51 years) 4.9 kg/m^2^

^c^For definitions see Table 1

### Disease severity

RF was a robust significant predictor of erosion score progression as well as for JSN score progression over 5 years (Tables 3 and 4).

Anti-CCP significantly predicted erosion and JSN score progression in crude models but was significant only for erosion score progression in the adjusted analyses (Tables 3 and 4).

Markers of inflammation significantly predicted both erosion and JSN score progression in crude models, but only ESR and not CRP were significant in the adjusted models (Tables 3 and 4).

While DAS28 analyzed as a continuous variable was not significantly associated with either erosion or JSN score progression in adjusted models, separate analysis of the baseline category of disease activity revealed a significantly increased risk of erosion score progression up to 5 years for patients with high baseline DAS28 (> 5.1) compared to those with low to moderate disease activity (Table 3).

No significant associations were observed in adjusted models for HAQ, swollen and tender joint counts, VAS for patients’ global assessment of disease activity or pain, or time from symptom onset to DMARD initiation, when analyzed as continuous variables, for either erosion or JSN score progression up to 5 years (Tables 3 and 4).

Analyzed as a continuous variable, COMP did not predict erosion or JSN score progression. When dichotomized using a predefined cut-off (> 12 units/L) [29], high level of COMP was significantly associated with erosion score progression in adjusted models, but not JSN score progression.

Baseline presence of erosions was significantly associated with erosion and JSN score progression up to 5 years in crude analyses, but only with erosion score progression in the adjusted models (Tables 3 and 4).

As for the baseline erosion and JSN scores respective ability to predict separate progression, erosion score at baseline predicted further erosion score progression in the crude model (Table 3) and also when adjusted for RF [*β* = 0.08 (95% CI 0.03, 0.12)], while JSN score did not. For JSN score progression, both erosion and JSN scores at baseline were significantly associated with further progression in crude models (Table 4) and when adjusted for RF and age [*β* = 0.11 (95% CI 0.05, 0.16), *β* = 0.10 (95% CI 0.04, 0.16)], respectively.

## Discussion

We confirmed several established and identified some novel predictors of radiographic progression. There was a reduced risk of JSN progression over 5 years among overweight or obese patients. In female patients, this effect was seen for older (> 51 years) but not younger women. Truncal fat was associated with reduced risk of JSN progression in women but not in men. Smoking was an independent predictor of erosion progression.

In the literature, RA associated autoantibodies are consistently associated with worse radiographical outcomes [6]. Similar to our results, in two studies of RA patients with longer disease duration, RF and ACPA were associated with more joint damage, in particular erosions [30, 31].

Systemic inflammation (measured by standard laboratory markers) has been shown to predict joint damage [6], but its specific effects on erosion and JSN development have been less studied. Similar to our results, Nawata et al. show baseline CRP levels to be related with both erosion and JSN progression [32].

Although some previous studies have shown COMP to be associated with levels of initial radiographic joint damage [33–35], results of studies evaluating radiographic damage over time are mixed [7–9, 33, 34, 36, 37]. Disease duration and timing of radiographic follow-up in available studies on COMP and radiographic damage are likely of importance, possibly explaining the above mixed results. Our results suggest a weak association for elevated baseline COMP with radiographic progression.

The development of erosions and JSN are considered partly separate but intertwined pathophysiological disease processes in RA. The timing of their development and whether each is a risk factor for progression of the other has been evaluated in a few studies. Consistent with our results, all available studies have shown that early JSN is predictive of future JSN progression [38–40]. Furthermore, most studies have shown early erosions to be associated with further erosion progression [38, 39]. Regarding the predictive value of early erosions for JSN and vice versa, results from previous studies are divergent [38–40]. In the present study, erosion score predicted JSN progression, while JSN was not a significant predictor of erosion progression—suggesting that erosions usually precede JSN in early RA.

Previous studies on smoking and radiographic progression have shown mixed results [2, 12, 13, 41–49]. In our previous study of prediction of total SHS progression [11] and especially in the present study, data suggest effects of smoking on radiographic progression and erosion progression in particular, that are independent of RF, disease activity, inflammation, and BMI. To our knowledge, the present study is the first to show independent associations of smoking with erosion progression but not JSN progression.

In a previous study, we have shown overweight or obesity to be associated with less rapid radiographic progression [11]. Similarly, several studies have consistently shown links between higher BMI and less radiographic joint damage, summarized in a systematic review by Vidal et al. [50]. In the present study, overweight or obesity was associated with less JSN progression, but not erosion progression. This is in line with a study by Alarcon et al., where higher BMI was associated more clearly with erosion-dominant than JSN-dominant pattern of joint damage [30], while BMI was negatively associated with both types joint damage in an earlier study by Hashimoto et al. [39]. We found that higher ratio of truncal fat was predictive of less JSN progression in women, while no effect was observed in men. To evaluate if age influences the effects of body constitution on JSN progression in women, we used the reported median age of natural menopause of 51 years [28] and stratified the analyses according to this cut off. The negative association between overweight or obesity and JSN progression was significant in the older (mainly post-menopausal) group, while no such effect was seen in the younger. To our knowledge, this is the first study providing indications of potential effects of interaction between menopausal age and BMI on the rate of JSN progression in RA. Whether physiological age-related changes in body constitution alter levels of adipokines important in the pathogenesis of destructive arthritis or if the effects are mediated by changes in sex hormones, other hormones, or chemokines, is unclear and should be further investigated.

Limitations in this study include the relatively small sample size, which affects statistical power for the multivariate analyses. Furthermore, the patients were included just before or shortly after the introduction of biologic DMARDs for the treatment of RA, and they were classified according to the 1987 ACR criteria. The results of this study may not apply to patients diagnosed according to more recent algorithms, in particular those with ready access to biologics who are treated according to a treat to target strategy [23]. Changes in smoking pattern, in particular passive smoking, over time could also influence the results.

Another limitation is that the scoring of the radiographs was performed by a single reader. As data on smoking, BMI, and COMP were only available at baseline, longitudinal evaluation of the impact of these factors was not possible. Data on body composition by DXA was only available for a subset of the full cohort.

Strengths of our study include the structured longitudinal follow-up of an inception cohort from a defined catchment area. Therefore, selection bias is not a major issue in this study, and the results could be generalized to patients with RA seen in clinical practice.

## Conclusion

Overweight RA patients had less JSN progression, independent of smoking status. This effect was seen in particular among older women (mainly post-menopausal), but not younger. Truncal fat was associated with less JSN progression in female patients. Smoking predicted erosion progression, and erosions may precede JSN. BMI and fat distribution may influence cartilage damage in early RA and might be related to hormonal factors.

## Data Availability

The datasets generated and/or analyzed during the current study are not publicly available due to Swedish legislation (the Personal Data Act), but a limited and fully anonymized dataset containing the individual patient data that support the main analyses is available from the corresponding author on reasonable request.

## Abbreviations

ACPA: Anti-citrullinated protein antibodies
ACR: American College of Rheumatology
Anti-CCP: Anti-citrullinated peptide antibodies
BFM: Body fat mass
BMD: Bone mineral density
BMI: Body mass index
CI: Confidence interval
COMP: Cartilage oligomeric matrix protein
CRP: C-reactive protein
DAS28: Disease activity score of 28 joints
DMARD: Disease-modifying anti-rheumatic drug
DXA: Dual energy x-ray absorptiometry
ESR: Erythrocyte sedimentation rate
HAQ: Health assessment questionnaire
ICC: Intra-class correlation coefficient
ICR: Interquartile range
JSN: Joint space narrowing
RA: Rheumatoid arthritis
RF: Rheumatoid factor
SD: Standard deviation
SHS: Modified Sharp/van der Heijde score
VAS: Visual analogue scale
WHO: World health organization

## References

[1] Combe B, Dougados M, Goupille P, Cantagrel A, Eliaou JF, Sibilia J (2001). Prognostic factors for radiographic damage in early rheumatoid arthritis: a multiparameter prospective study. Arthritis Rheum.

[2] Forslind K, Ahlmen M, Eberhardt K, Hafstrom I, Svensson B, Group BS (2004). Prediction of radiological outcome in early rheumatoid arthritis in clinical practice: role of antibodies to citrullinated peptides (anti-CCP). Ann Rheum Dis.

[3] Visser K, Goekoop-Ruiterman YP, de Vries-Bouwstra JK, Ronday HK, Seys PE, Kerstens PJ (2010). A matrix risk model for the prediction of rapid radiographic progression in patients with rheumatoid arthritis receiving different dynamic treatment strategies: post hoc analyses from the BeSt study. Ann Rheum Dis.

[4] Fautrel B, Granger B, Combe B, Saraux A, Guillemin F, Le Loet X (2012). Matrix to predict rapid radiographic progression of early rheumatoid arthritis patients from the community treated with methotrexate or leflunomide: results from the ESPOIR cohort. Arthritis Res Ther.

[5] Tobon G, Saraux A, Lukas C, Gandjbakhch F, Gottenberg JE, Mariette X (2013). First-year radiographic progression as a predictor of further progression in early arthritis: results of a large national French cohort. Arthritis Care Res (Hoboken).

[6] Carpenter L, Nikiphorou E, Sharpe R, Norton S, Rennie K, Bunn F, et al. Have radiographic progression rates in early rheumatoid arthritis changed? A systematic review and meta-analysis of long-term cohorts. Rheumatology (Oxford). 2016. 10.1093/rheumatology/kew004. 10.1093/rheumatology/kew004 26961746

[7] Forslind K, Eberhardt K, Jonsson A, Saxne T (1992). Increased serum concentrations of cartilage oligomeric matrix protein. A prognostic marker in early rheumatoid arthritis. Br J Rheumatol.

[8] Skoumal M, Kolarz G, Klingler A (2003). Serum levels of cartilage oligomeric matrix protein. A predicting factor and a valuable parameter for disease management in rheumatoid arthritis. Scand J Rheumatol.

[9] Lindqvist E, Eberhardt K, Bendtzen K, Heinegard D, Saxne T (2005). Prognostic laboratory markers of joint damage in rheumatoid arthritis. Ann Rheum Dis.

[10] Andersson ML, Svensson B, Petersson IF, Hafstrom I, Albertsson K, Forslind K (2013). Early increase in serum-COMP is associated with joint damage progression over the first five years in patients with rheumatoid arthritis. BMC Musculoskelet Disord.

[11] Rydell E, Forslind K, Nilsson JA, Jacobsson LTH, Turesson C (2018). Smoking, body mass index, disease activity, and the risk of rapid radiographic progression in patients with early rheumatoid arthritis. Arthritis Res Ther.

[12] Saevarsdottir S, Rezaei H, Geborek P, Petersson I, Ernestam S, Albertsson K (2015). Current smoking status is a strong predictor of radiographic progression in early rheumatoid arthritis: results from the SWEFOT trial. Ann Rheum Dis.

[13] de Rooy DP, van Nies JA, Kapetanovic MC, Kristjansdottir H, Andersson ML, Forslind K (2014). Smoking as a risk factor for the radiological severity of rheumatoid arthritis: a study on six cohorts. Ann Rheum Dis.

[14] Kaufmann J, Kielstein V (2003). Relation between body mass index and radiological progression in patients with rheumatoid arthritis. J Rheumatol.

[15] Westhoff G, Rau R, Zink A (2007). Radiographic joint damage in early rheumatoid arthritis is highly dependent on body mass index. Arthritis Rheum.

[16] Baker JF, Ostergaard M, George M, Shults J, Emery P, Baker DG (2014). Greater body mass independently predicts less radiographic progression on X-ray and MRI over 1-2 years. Ann Rheum Dis.

[17] Giles JT, Allison M, Bingham CO, Scott WM, Bathon JM (2009). Adiponectin is a mediator of the inverse association of adiposity with radiographic damage in rheumatoid arthritis. Arthritis Rheum.

[18] Meyer M, Sellam J (2013). Serum level of adiponectin is a surrogate independent biomarker of radiographic disease progression in early rheumatoid arthritis: results from the ESPOIR cohort. Arthritis Res Ther.

[19] Boini S, Guillemin F (2001). Radiographic scoring methods as outcome measures in rheumatoid arthritis: properties and advantages. Ann Rheum Dis.

[20] Smolen JS, Aletaha D, Barton A, Burmester GR, Emery P, Firestein GS (2018). Rheumatoid arthritis. Nat Rev Dis Primers.

[21] Arnett FC, Edworthy SM, Bloch DA, McShane DJ, Fries JF, Cooper NS (1988). The American rheumatism association 1987 revised criteria for the classification of rheumatoid arthritis. Arthritis Rheum.

[22] Ekdahl C, Eberhardt K, Andersson SI, Svensson B (1988). Assessing disability in patients with rheumatoid arthritis. Use of a Swedish version of the Stanford Health Assessment Questionnaire. Scand J Rheumatol.

[23] Smolen JS, Aletaha D, Bijlsma JW, Breedveld FC, Boumpas D, Burmester G (2010). Treating rheumatoid arthritis to target: recommendations of an international task force. Ann Rheum Dis.

[24] Geborek P, Nitelius E, Noltorp S, Petri H, Jacobsson L, Larsson L (2005). Population based studies of biological antirheumatic drug use in southern Sweden: comparison with pharmaceutical sales. Ann Rheum Dis.

[25] Book C, Karlsson MK, Akesson K, Jacobsson LT (2009). Early rheumatoid arthritis and body composition. Rheumatology (Oxford).

[26] van der Heijde D (2000). How to read radiographs according to the Sharp/van der Heijde method. J Rheumatol.

[27] Forslind K, Svensson B (2019). Repair of bone erosions in rheumatoid arthritis: a systematic literature review. Scand J Rheumatol.

[28] Hagstad A (1988). Gynecology and sexuality in middle-aged women. Women Health.

[29] Turesson C, Bergstrom U, Jacobsson LT, Truedsson L, Berglund G, Saxne T (2011). Increased cartilage turnover and circulating autoantibodies in different subsets before the clinical onset of rheumatoid arthritis. Ann Rheum Dis.

[30] Alarcon RT, Fernandes Ada R, Laurindo IM, Bertolo MB, Pinheiro GC, Andrade LE (2015). Characterization of cumulative joint damage patterns in patients with rheumatoid arthritis: a clinical, serological, and gene polymorphism perspective. J Rheumatol.

[31] Syversen SW, Goll GL, van der Heijde D, Landewe R, Lie BA, Odegard S (2010). Prediction of radiographic progression in rheumatoid arthritis and the role of antibodies against mutated citrullinated vimentin: results from a 10-year prospective study. Ann Rheum Dis.

[32] Nawata M, Saito K, Fukuyo S, Hirata S, Tanaka Y. Clinically relevant radiographic progression in joint destruction in RA patients with abnormal MMP-3 or high levels of CRP despite 1-year treatment with infliximab. Mod Rheumatol. 2016;26:807–12. 10.3109/14397595.2016.1158386 26915532

[33] Fex E, Eberhardt K, Saxne T (1997). Tissue-derived macromolecules and markers of inflammation in serum in early rheumatoid arthritis: relationship to development of joint destruction in hands and feet. Br J Rheumatol.

[34] de Jong Z, Munneke M, Vilim V, Zwinderman AH, Kroon HM, Ronday HK (2008). Value of serum cartilage oligomeric matrix protein as a prognostic marker of large-joint damage in rheumatoid arthritis--data from the RAPIT study. Rheumatology (Oxford).

[35] Fujikawa K, Kawakami A, Tamai M, Uetani M, Takao S, Arima K (2009). High serum cartilage oligomeric matrix protein determines the subset of patients with early-stage rheumatoid arthritis with high serum C-reactive protein, matrix metalloproteinase-3, and MRI-proven bone erosion. J Rheumatol.

[36] den Broeder AA, Joosten LA, Saxne T, Heinegard D, Fenner H, Miltenburg AM (2002). Long term anti-tumour necrosis factor alpha monotherapy in rheumatoid arthritis: effect on radiological course and prognostic value of markers of cartilage turnover and endothelial activation. Ann Rheum Dis.

[37] Syversen SW, Goll GL, van der Heijde D, Landewe R, Gaarder PI, Odegard S (2009). Cartilage and bone biomarkers in rheumatoid arthritis: prediction of 10-year radiographic progression. J Rheumatol.

[38] Smolen JS, van der Heijde DM, Aletaha D, Xu S, Han J, Baker D (2009). Progression of radiographic joint damage in rheumatoid arthritis: independence of erosions and joint space narrowing. Ann Rheum Dis.

[39] Hashimoto J, Garnero P, van der Heijde D, Miyasaka N, Yamamoto K, Kawai S (2009). A combination of biochemical markers of cartilage and bone turnover, radiographic damage and body mass index to predict the progression of joint destruction in patients with rheumatoid arthritis treated with disease-modifying anti-rheumatic drugs. Mod Rheumatol.

[40] Landewe R, Smolen JS, Florentinus S, Chen S, Guerette B, van der Heijde D (2015). Existing joint erosions increase the risk of joint space narrowing independently of clinical synovitis in patients with early rheumatoid arthritis. Arthritis Res Ther.

[41] Saag KG, Cerhan JR (1997). Cigarette smoking and rheumatoid arthritis severity. Ann Rheum Dis.

[42] Wolfe F (2000). The effect of smoking on clinical, laboratory, and radiographic status in rheumatoid arthritis. J Rheumatol.

[43] Mattey DL, Hutchinson D, Dawes PT, Nixon NB, Clarke S, Fisher J (2002). Smoking and disease severity in rheumatoid arthritis: association with polymorphism at the glutathione S-transferase M1 locus. Arthritis Rheum.

[44] Papadopoulos NG, Alamanos Y, Voulgari PV, Epagelis EK, Tsifetaki N, Drosos AA (2005). Does cigarette smoking influence disease expression, activity and severity in early rheumatoid arthritis patients?. Clin Exp Rheumatol.

[45] Manfredsdottir VF, Vikingsdottir T, Jonsson T, Geirsson AJ, Kjartansson O, Heimisdottir M (2006). The effects of tobacco smoking and rheumatoid factor seropositivity on disease activity and joint damage in early rheumatoid arthritis. Rheumatology (Oxford).

[46] Westhoff G, Rau R, Zink A (2008). Rheumatoid arthritis patients who smoke have a higher need for DMARDs and feel worse, but they do not have more joint damage than non-smokers of the same serological group. Rheumatology (Oxford).

[47] Ruiz-Esquide V, Gomez-Puerta JA, Canete JD, Graell E, Vazquez I, Ercilla MG (2011). Effects of smoking on disease activity and radiographic progression in early rheumatoid arthritis. J Rheumatol.

[48] Vesperini V, Lukas C, Fautrel B, Le Loet X, Rincheval N, Combe B (2013). Association of tobacco exposure and reduction of radiographic progression in early rheumatoid arthritis: results from a French multicenter cohort. Arthritis Care Res (Hoboken).

[49] Haye Salinas MJ, Retamozo S, Alvarez AC, Maldonado Ficco H, Dal Pra F, Citera G (2015). Effects of cigarette smoking on early arthritis: a cross-sectional study-data from the Argentine Consortium for Early Arthritis (CONAART). Rheumatol Int.

[50] Vidal C, Barnetche T, Morel J, Combe B, Daien C (2015). Association of body mass index categories with disease activity and radiographic joint damage in rheumatoid arthritis: a systematic review and Metaanalysis. J Rheumatol.

